# ROUX-IN-Y GASTROJEJUNAL BYPASS: WHICH ANESTHETIC TECHNIQUE HAS BEST RESULTS?

**DOI:** 10.1590/0102-672020200002e1530

**Published:** 2021-05-14

**Authors:** Arthur RUZZON, Paulo Afonso Nunes NASSIF, Lais PRIGOL, Lucas BUZO, Guilherme WENDLER, Eduardo WENDLER, Ilana Barrichello Torres WENDLER, Igor RUZZON, Caio Henrique Marchette GOVEIA, Lucas Augusto Prestes GONÇALVES

**Affiliations:** 1 Postgraduate Program in Principles of Surgery, Mackenzie Evangelical College of Paraná/Medical Research Institute, Curitiba, PR, Brazil; 2 Rocio Hospital, Campo Largo, PR, Brazil

**Keywords:** Obesity, Bariatric, Pain, ERAS, Recovery, Opioid, Obesidade, Bariátrica, Dor, ERAS, Recuperação, Opioide

## Abstract

**Background:**

As the number of bariatric operations increases, there is a greater interest in knowledge, experience and skills in the operative and anesthetic management of obese people. Anesthetic recovery is an important point in the therapeutic approach and less adverse effects delaying discharge of these patients are necessary to be kept in mind by the surgical team.

**Aim:**

To compare anesthetic-analgesic techniques in the opioid-sparing era through epidural administration of local anesthetic associated with low-dose morphine vs. clonidine and analyze the impact of analgesia on the effectiveness of postoperative recovery by comparing these two techniques.

**Methods:**

Randomized, double-blind clinical trial with 66 patients candidates for Roux-en-Y gastrojejunal bypass divided into two groups: morphine group and clonidine group. Multimodal analgesia included epidural anesthesia with 0.375% ropivacaine 20 ml at the eighth thoracic vertebra with the association of morphine (morphine group) at a dose of 15 mcg / kg or clonidine (clonidine group) at a dose of 1 mcg / kg.

**Results:**

The groups were homogeneous and statistical significance was found when analyzing the difference in pain between them in the first postoperative period. The pain was higher in the clonidine group, as in this period, analgesic rescue was also better in this group. In the other times, there was no significance in the differences regarding pain and rescue. The return of intestinal motility in the morphine group was earlier in the first postoperative period. Nausea, vomiting and hospital discharge did not show significant differences between groups.

**Conclusion:**

Epidural anesthesia with low-dose morphine allowed less pain during the entire hospital stay, with a positive impact on patient recovery.

## Graphical abstract

[Fig f1a]

**Figure f1a:**
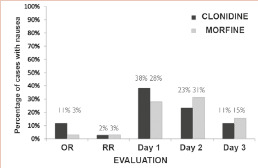
Nausea and vomiting groups x moments

[Fig f1as]

**Figure f1as:**
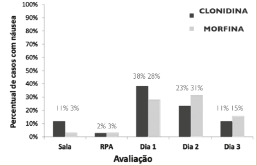
Náuseas e vômitos grupos x momentos

## RESUMO

**Racional:**

Com o aumento do número de operações bariátricas torna-se maior o interesse pelo conhecimento, experiência e habilidades no manejo operatório e anestésico de obesos. A recuperação anestésica é ponto importante na abordagem terapêutica e menos efeitos adversos que retardem a alta são necessários estar em mente da equipe cirúrgica.

**Objetivo:**

Comparar técnicas anestésico-analgésicas na era poupadora de opioides através da administração epidural de anestésico local associado à morfina em baixa dose vs. à clonidina e analisar o impacto da analgesia na efetividade da recuperação pós-operatória comparando as duas técnicas.

**Métodos:**

Ensaio clínico randomizado e duplo-cego com 66 pacientes candidatos ao bypass gastrojejunal em Y-de-Roux divididos em dois grupos: grupo morfina e grupo clonidina. A analgesia multimodal incluiu na anestesia epidural com ropivacaína 0,375% 20 ml na altura da oitava vértebra torácica a associação de morfina (grupo morfina) em dose de 15 mcg/kg ou de clonidina (grupo clonidina) na dose 1 mcg/kg.

**Resultados:**

Os grupos se mostraram homogêneos e foi encontrada significância estatística ao analisar a diferença da dor entre eles no primeiro pós operatório. A dor foi superior no grupo clonidina, como também neste período o resgate analgésico foi melhor neste grupo. Nos demais tempos não houve significância nas diferenças quanto à dor e resgate. O retorno da motilidade intestinal no grupo morfina foi mais precoce no primeiro pós-operatório. Náuseas, vômitos e a alta hospitalar não apresentaram diferenças significativas entre os grupos.

**Conclusão:**

A anestesia epidural com morfina em baixa dose permitiu menor dor durante todo internamento com impacto positivo em relação a recuperação dos pacientes.

## INTRODUCTION

Obesity is defined as a metabolic disease in which the accumulation of adipose tissue has a proportion of body mass higher than normal, consisting of the fastest growing disease in Brazil and establishing itself as a major health problem^8^.According to the Brazilian Association for the Study of Obesity and Metabolic Syndrome, the data reveal that more than 50% of the Brazilian population is overweight and in children the prevalence is 15%^17^. In the Unified Health System of Brazil (SUS) the number of bariatric operations increased, only between 2008 and 2017 it grew 215%, according to data from the Brazilian Society of Bariatric and Metabolic Surgery^1^.

With the growing obesity epidemic, and a consequent increase in the number of procedures for its treatment and control, there is a greater interest in acquiring knowledge, experience and skills for the surgical and anesthetic management of these patients^10^ ^,^ ^21^. These involve several factors, from measures for postoperative analgesia, presence of nausea, vomiting and effective peristalsis, which contribute to lower morbidity and mortality and early discharge from hospital^4^.

Postoperative pain still persists as a significant and omnipresent adversity. It, at the same time, is associated with a myriad of postoperative complications that lead to delayed recovery and consequently delayed hospital discharge^7^.

Da Silva and Ribeiro (2011)^5^ emphasize that pain in obese individuals is even more important due to the increased risk of respiratory and gastrointestinal complications. Both the diaphragmatic irritation caused by the use of the retractor pulled in laparotomic gastroplasty and the pneumoperitoneum in the laparoscopic route, are the factors that most lead to immediate postoperative pain, even in the operating room^7^.

With the use of opioids for pain management in large abdominal procedures - as proposed for this study - side effects such as nausea and vomiting, paralytic ileus, abdominal distension, urinary retention and consequent delay in hospital discharge, are expected.

In the opioid-sparing era, we seek to optimize this recovery by reducing side effects with adequate analgesia and early discharge^21^. Better results are sought with techniques that combine local anesthetic in epidural administration. The Enhanced Recovery after Surgery (ERAS) mentions that there are few studies on analgesic efficacy comparing adjuvants on the topic^2^ ^,^ ^19^ ^,^ ^20^.

The objective of this clinical trial was to analyze the impact of analgesia on the effectiveness of postoperative recovery by comparing anesthetic technique with the use of neuroaxial opioids in reduced dose vs. opioid-free neuroaxial technique.

## METHODS

In this randomized, double-blind clinical trial, 66 candidate patients for Roux-en-Y gastric bypass (RYGB) were recruited. It was approved by the Research Ethics Committee of Faculdade Evangélica Mackenzie do Paraná - CEP/FEMPAR and according to the attributions defined in Resolution 466/12 CNS under opinion number 3,466,603.

After applying the inclusion and exclusion criteria, patients were randomized and divided into two groups: morphine group (MG) and clonidine group (CG). The operations were performed in a single institution - Hospital do Rocio, Campo Largo, PR, Brazil.

Those who agreed to participate in the study were included by signing the informed consent form, and were elected on an outpatient basis by the surgical team for the proposed operation because they had a BMI >35 kg/m^2^ associated with hypertension and/or diabetes or a BMI >40 kg/m^2^. Those with a history of postoperative nausea and vomiting, renal or hepatic insufficiency or dysfunction, coagulation disorders, heart disease and dipyrone and/or non-hormonal anti-inflammatory drugs allergy were excluded.

Patients were randomly assigned to both groups. Randomization was performed using the Random^®^ program, in which the number provided corresponded to the group in which each patient fit. The RYGB surgical technique consists of a small gastric pouch (30 ml) created from an upper gastric area anastomosed to the small intestine that was sectioned 120 cm distal to the duodenojejunal flexure, creating a biliopancreatic and alimentary loop. The distal biliopancreatic segment was connected to the food loop, approximately 120 cm from the gastrojejunal anastomosis.

Anesthesia was standardized for both groups, with anesthetic induction with propofol 1.5 mg/kg (ideal weight), fentanyl 3 mcg/kg (ideal weight) and cisatracurium 0.15 mg/kg (ideal weight). Mechanical ventilation was used protectively with a tidal volume between 6-8 ml/kg (ideal weight), using an oxygen concentration of 30-50% with compressed air. The volume replacement therapy used was conservative with 15 ml/kg of Ringer-Lactate. Conventional monitoring included: cardioscopy with five leads, non-invasive blood pressure, pulse oximetry, anesthetic depth monitor performed with bispectral index (BIS) aiming at a target of 40-60, to ensure adequate hypnotic effect in general anesthesia and monitoring of neuromuscular block performed through “Train of four”(TOF), maintaining the value of 0 (zero) during the operation with TOF extubation criterion >0.9. Anesthetic maintenance was performed with sevoflurane, varying the expired fraction between 1.5-2.5%, according to the BIS target.

The proposed analgesia was multimodal including dipyrone 2 g, ketorolac 30 mg and epidural anesthesia at the eighth thoracic vertebra (T8) with 0.375% ropivacaine 20 ml associated with morphine (MG) at a dose of 15 mcg/kg (real weight), whereas clonidine (CG) was at a dose of 1 mcg/kg (real weight). General anesthesia associated with epidural block was performed with the analgesic medication proposed for each group and with proper identification in the patient file. The prevention of nausea and vomiting was carried out with ondansetrone 8 mg, dexamethasone 4 mg and lizapride 50 mg. The analgesic rescue was performed sequentially with tramadol 100 mg in 100 ml of 0.9% saline up to 8/8 h, and in case of non-resolution of the pain, intravenous doses of morphine (0.05 mg/kg) could administered up to 4/4 h for control.

After discharge from the post-anesthetic recovery room, patients were prescribed and medicated with ketorolac 30 mg every 8 h, dipyrone 2 g every 6 h and rescues as mentioned above with tramadol and morphine for those with Numerical Verbal Scale (NVS) >4. Prophylaxis of nausea and vomiting was performed intravenously with ondansetron 8 mg every 8 h and, if necessary, rescues was made with alizapride 50 mg until 8/8 h.

The NVS scale was used for pain assessments, explaining to the patient that it could vary from 0 to 10, with extremes 0 being no pain and 10 being the worst possible sensation of pain. As well as the presence of nausea or vomiting, and the need for analgesic rescues was also assessed. Through the questionnaire “Douleur neuropathique 4 questions” (DN4) the score was analyzed, being considered pain with neuropathic characteristic if added 4 or more points. The return of intestinal function was analyzed through the presence of flatus and the absence of abdominal distensions associated with nausea and vomiting.

The evaluation of the patients was performed by a second anesthesiologist, who was unaware of the medication used in each patient, as well as, which group belonged in the periods of immediate postoperative recovery, in the postoperative recovery, and in the 1^st^, 2^nd^ and 3^rd^ postoperative (PO) day. After discharge of the recovery room, pain was also investigated regarding its neuropathic character through the DN4 questionnaire and return of intestinal motility.

### Statistical analysis

For the description of the quantitative variables, the statistics of mean, median, 1^st^ and 3^rd^ quartiles, minimum and maximum values and standard deviation were considered. To summarize the qualitative variables, frequencies and percentages were presented. For comparison of the two groups, in relation to quantitative variables, Student’s t-tests for independent samples and non-parametric Mann-Whitney samples were used. Regarding qualitative variables, comparisons were made using Fisher’s exact test. For comparison of the moments, within each group, in relation to pain assessment, Friedman’s nonparametric test was considered. Values of p <0.05 indicated statistical significance. The data were analyzed using the IBM SPSS Statistics v.20.0 computer program. Armonk, NY: IBM Corp.

## RESULTS

The sample of this clinical trial consisted of 66 patients, 34 from the CG and 32 from the MG. Table 1 shows the homogeneity between the groups regarding weight, height, age and BMI (Table 1).

**TABLE 1:** Homogeneity of groups

Variable	Group	n	Mean	Median	Minimum	Maximum	SD	p*
Weight	C	34	112.3	112.0	81.0	148.0	15.9	0.702
	M	32	114.3	108.5	74.0	180.0	24.2	
Height	C	34	1.6	1.6	1.4	1.8	0.1	0.488
	M	32	1.6	1.6	1.4	1.8	0.1	
Age	C	34	37.0	35.5	21.0	63.0	10.3	0.152
	M	32	40.6	40.5	25.0	59.0	10.0	
BMI	C	34	43.9	42.7	35.7	61.4	5.1	0.821
	M	32	43.5	43.5	35.7	70.3	6.7	

(*) Student’s t test for independent samples; p <0.05; SD=standard deviation; C = Clonidine; M = Morphine

### Pain

In the descriptive statistics of the pain assessment, it was noted that in the odd group the highest average score (4.6) occurred in the 1^st^ PO, decreasing to an average of 1.4 points in the 3^rd^ PO; in the operating room and in the recovery room it was 0 and close to 0, respectively (Table 2). In the quantification of pain in the operating room, compared to the moments from 1^st^ PO to 3^rd^ PO, a significant difference was found with p<0.001 between the calculated values, showing the absence of pain upon awakening, but with the appearance of significant pain in subsequent days (Table 3).

**TABLE 2:** Clonidine group pain (CG)

Moment	n	Mean	Min	1st quartile	Median	3rd quartile	Max	p*
OR	34	0.0	0	0	0	0	0	
RR	34	0.4	0	0	0	0	7	
Day 1	34	4.6	0	0.75	5	8	9	<0.001
Day 2	34	3.1	0	0.25	3	5	8	
Day 3	34	1.4	0	0	0	2	6	

Moments under comparison	p
Operating Room x Day 1	< 0,001
Operating Room x Day 2	< 0,001
Operating Room x Day 3	< 0,001

* =Kruskal-Wallis non-parametric test; p<0,05; OR=operating room; RR=recovery room; Min - Minimum; Max = Maximum

In the MG analysis, mean pain scores ranging from 1.3 points in the operating room and 1^st^ PO, 1.6 points in the recovery room and 2 in the PO decreased to an average of 0.3 points in the 3^rd^ PO (Table 3). When comparing the means between the moments, no significant differences were observed through statistical tests.

**TABLE 3:** Morphine group pain

Moment	n	Mean	Min	1st quartile	Median	3rd quartile	Max	p*
OR	32	1.3	0	0	0	0	10	
RR	32	1.6	0	0	0	3	10	
Day 1	32	1.3	0	0	0	2	8	0.042
Day 2	32	1.6	0	0	0	3	8	
Day 3	32	0.3	0	0	0	0	3	

* =Kruskal-Wallis non-parametric test; p<0,05; OR=operating room; RR=recovery room

When comparing the groups at different times, it was possible to notice p <0.001 when analyzing the difference in pain scores in the 1^st^ PO, in which the averages were 4.6 in the CG and 1.3 points in the MG. When comparing the two groups in the two rooms, although the CG had an average equal to 0 and close to 0 two times and the MG averages greater than 1, there was no significance in the expression of these values (Table 4).

**TABLE 4:** Analysis between pain groups at different times

Moment	Group	n	Mean	Min	1st quartile	Median	3rd quartile	Max	p*
OR	C	34	0.0	0	0	0	0	0	0.191
	M	32	1.3	0	0	0	0	10	
RR	C	34	0.4	0	0	0	0	7	0.101
	M	32	1.6	0	0	0	3	10	
Day 1	C	34	4.6	0	0.75	5	8	9	<0.001
	M	32	1.3	0	0	0	2	8	
Day 2	C	34	3.1	0	0.25	3	5	8	0.014
	M	32	1.6	0	0	0	3	8	
Day 3	C	34	1.4	0	0	0	2	6	0.023
	M	32	0.3	0	0	0	0	3	

* =Kruskal-Wallis non-parametric test; p<0,05; OR=operating room; RR=recovery room; C = Clonidine; M = Morphine

### Pain relief

In the analysis of the needs for analgesic rescues, it was noted that in the two rooms there was no significant difference in the use of tramadol, as occurred in the 2^nd^ PO and the 3^rd^ PO. However, in the 1^st^ PO the CG needed 38.2% of analgesics compared to only 6.3% in the MG (p=0.003).

### Nausea and vomiting

**FIGURE 1:**
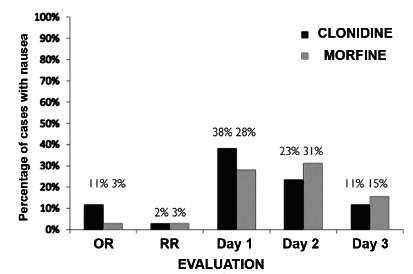
Nausea and vomiting groups x moments

When assessing the presence of nausea and vomiting in both groups at the moments studied, no significance was found in the differences found (Figure 1).

OR=operating room; RR=Recovery room

### Return of gastrointestinal function

It was observed that the MG showed an earlier gastrointestinal motility return (1^st^ PO) in 12.5% vs. 0% in the CG (p=0.05) without statistical significance, as well as the other analyzes between the other moments in the groups (Figure 2).

**FIGURE 3:**
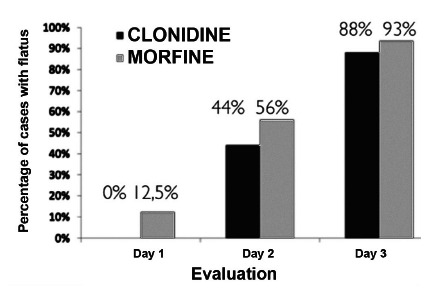
Return of gastrointestinal function

### Hospital discharge

Hospital discharge in both groups occurred at the end of the 3^rd^ PO, and all 66 patients had satisfactory criteria for doing so.

### Neuropathic pain

When tracking neuropathic pain in both groups at different times, there was no pain in any of the groups scoring through DN4 that characterized them as neuropathic.

## DISCUSSION

When analyzing pain in order to be able to assess the impact that analgesia can have on the recovery of patients undergoing RYGB, the concern related to the global fever of opioid abuse is expressed^11^. At the same time that inadequate pain control prevents postoperative rehabilitation, reduces quality of life and causes personal wear, it also contributes to the increase in national health expenditure.

The primary class of drugs used as antinociceptive agents are opioids, and they target multiple classes of receptors in the periaqueductal gray matter, spinal cord, amygdala, rostral ventral cord and cortex^3^.

Opioids continue to play an important role in controlling postoperative pain; however, they are not exempt from side effects, and a multimodal approach has been suggested to improve postoperative analgesia and, at the same time, mitigate these side effects^14^.

In morbidly obese, the pharmacokinetics of morphine are comparable to that of healthy volunteers; therefore, no dose adjustment based on weight is necessary according to studies by Hoogd et al. (2017)^6^.

As an alternative to opioids, alpha-2 agonists are available, commonly used in clinical practice such as clonidine and dexmedetomedine. Graff and Groser (2018)^11^ reported that the primary mechanism of antinociception is the direct stimulation of alpha-2 adrenoreceptors in the central nervous system including the spinal cord, inhibiting nociceptive neuronal activation, reducing the release of substance P responsible for painful responses. These same authors also confirm that this drug class can significantly reduce the consumption of opioids, nausea and vomiting in the postoperative period, anxiety, postoperative tremors and responses to stress during the operation.

However, when comparing the two groups studied in this study, it was noted that the clonidine associated with local anesthetic in epidural anesthesia, had satisfactory pain control only in the initial postoperative recovery, that is , in OR and RR, showing a significant statistical difference when compared to the lowest mean of pain with the group that received low dose morphine in the 1^st^ PO and 2^nd^ PO; consequently, it required a higher frequency of analgesic rescues at these times, however significant only in the 1^st^ PO. It should be noted that in our analgesic technique we do not use epidural catheters for continued infusions.

In the study by Manion and Brennann (2011)^13^ it is mentioned that local anesthetics are usually combined with opioids in epidural anesthesia in order to provide an additive or synergistic analgesia, reducing the adverse effects related to the dose of one or the other isolated drug, combination of local anesthetics in the thoracic epidural route with opioids produces superior analgesia compared to the use of opioids or local anesthetics alone.

And so, it was confirmed, although with a moderate quality of evidence, the systematic review of Cochrane 2016^12^, containing 35 trials with 2731 participants, saying that an epidural anesthesia containing a mixture of local anesthetic with opioids will reduce pain on movement within 24 h after abdominal surgery, whether open or laparoscopic.

Regarding the presence of nausea and vomiting, a decrease in the incidence in the Odd group was expected, as mentioned above; however, at all times there were no differences with statistical significance, and it was also noted that in periods when the CG most needed pain relief with tramadol - a weak opioid - there was also no significant increase in the presence of nausea and vomiting.

In another systematic review of Cochrane 2016^18^, containing 22 clinical trials with 1138 participants, the researchers found high-quality evidence suggesting that epidural anesthesia containing local anesthetic with or without the addition of opioid decreases the time required for gastrointestinal transit to return. This is what happened in this study, since both groups had a return of intestinal motility mostly until the 2^nd^ PO; however, the group that received morphine, no matter how significant, started to show this return of motility earlier than those who received clonidine.

In the study by Rosen et al. (2018)^15^ showed that, in the context of multimodal analgesia following the ERAS protocol, the use of epidural anesthesia had no impact on the length of stay or consumption of opioids in-hospital after colorectal surgeries, as we noted in our study that the patients in both groups had the same hospital discharge time due to their aptitude for this, with average pain without significant differences, as well as the presence of a return of gastrointestinal motility, confirming the finding of the Cochrane systematic review (2016)^12^ in which there was very low quality evidence, showing that an epidural anesthesia containing a local anesthetic could reduce the hospital stay for open surgery.

Fonseca, Gatto and Tondatto (2016)^9^ reported that the association between the intensity of acute postoperative pain and the subsequent development of chronic pain was observed after some types of surgery, but it is still not completely clear whether this association is an indication of the extent of changes in neuroplasticity induced by the operation, due to the lack of adequate analgesia or that there may be preoperative predisposing factors. Another study by Sansone et al. (2015)^16^ showed that severe pain during the first 24 h after surgery seems to be a predictor of chronic post-surgical pain. And then, when we tracked signs of these neuropathic features through the DN4 questionnaire, we did not find scores that characterized this type of pain in any of the 66 patients.

## CONCLUSION

Comparing the two techniques, it can be concluded that epidural anesthesia containing low-dose morphine, compared to that with clonidine, showed uniformity of low pain indexes throughout hospitalization, causing a positive impact in relation to the recovery of patients undergoing RYGB.
